# Physicochemical Evolution and Molecular Adaptation of the Cetacean Osmoregulation-related Gene UT-A2 and Implications for Functional Studies

**DOI:** 10.1038/srep08795

**Published:** 2015-03-12

**Authors:** Jingzhen Wang, Xueying Yu, Bo Hu, Jinsong Zheng, Wuhan Xiao, Yujiang Hao, Wenhua Liu, Ding Wang

**Affiliations:** 1 Key Laboratory of Aquatic Biodiversity and Conservation of the Chinese Academy of Sciences; Institute of Hydrobiology, Chinese Academy of Sciences, Wuhan, Hubei 430072, China; 2 Marine Biology Institute, Shantou University, Shantou, Guangdong 515063, China; 3 University of Chinese Academy of Sciences, Beijing 100039, China

## Abstract

Cetaceans have an enigmatic evolutionary history of re-invading aquatic habitats. One of their essential adaptabilities that has enabled this process is their homeostatic strategy adjustment. Here, we investigated the physicochemical evolution and molecular adaptation of the cetacean urea transporter UT-A2, which plays an important role in urine concentration and water homeostasis. First, we cloned UT-A2 from the freshwater Yangtze finless porpoise, after which bioinformatics analyses were conducted based on available datasets (including freshwater baiji and marine toothed and baleen whales) using MEGA, PAML, DataMonkey, TreeSAAP and Consurf. Our findings suggest that the UT-A2 protein shows folding similar to that of dvUT and UT-B, whereas some variations occurred in the functional S_o_ and S_i_ regions of the selectivity filter. Additionally, several regions of the cetacean UT-A2 protein have experienced molecular adaptations. We suggest that positive-destabilizing selection could contribute to adaptations by influencing its biochemical and conformational character. The conservation of amino acid residues within the selectivity filter of the urea conduction pore is likely to be necessary for urea conduction, whereas the non-conserved amino acid replacements around the entrance and exit of the conduction pore could potentially affect the activity, which could be interesting target sites for future mutagenesis studies.

It is well known that cetaceans have a unique evolutionary history. They originated from terrestrial tetrapod mammals^1^,^2^,^3^,^4^, and were eventually distributed into aquatic environments after experiencing a period of semi-aquatic living^5^,^6^,^7^,^8^,^9^. Therefore, they have developed unique adaptive osmoregulatory strategies to maintain internal homeostasis. Osmoregulation in marine mammals, including cetaceans, has been of interest to physiologists for over a century^10^,^11^,^12^,^13^,^14^. Previous research indicates that three main factors facilitate this osmoregulation: 1) reducing skin and respiratory system water loss^15^,^16^,^17^,^18^,^19^; 2) obtaining water from food, seawater and their body fat^10^,^11^,^14^,^20^,^21^,^22^; and 3) concentrating urine and reabsorbing water^10^,^11^,^23^,^24^,^25^. All of these strategies are physiological mechanisms for water and electrolyte regulation that allow cetaceans to maintain a relatively stable and constant internal state in the face of environmental changes.

One of the most fundamental and efficient osmoregulation mechanisms is the capability of the kidneys to produce concentrated or diluted urine to match the intake and elimination of water and electrolytes^26^. In regard to the urine-concentrating mechanism, any hypothesis regarding the mechanism by which the inner medulla concentrates urine needs to include a urea effect^26^,^27^. As a crucial factor in osmolytic balance of the blood and urine^28^,^29^,^30^,^31^,^32^, urea can benefit water re-absorption in mammals^26^,^33^,^34^,^35^. Cloning of the protein urea transporter (UT, also known as solute carrier family 14, SLC14), which mediates urea conduction, has provided additional insight into the urine-concentrating mechanism^28^. The UT, which is located in the descending limbs of the loop of Henle and the inner medullary collecting ducts, facilitates urea accumulation in the renal medulla and equilibrates the urea concentrations between the urine and the interstitium, thereby influencing water re-absorption in the kidney^29^,^31^,^35^,^36^,^37^,^38^. Previous animal experiments have also verified the essential roles of UTs in animal homeostasis regulation. For example, mice lacking UTs cannot efficiently conserve water^39^,^40^,^41^ and mutant UTs in humans generate bladder cancer^42^,^43^ and create blood pressure problems^44^. Cetaceans usually consume lots of protein, which will generate large quantities of urea by catabolism. Therefore, cetaceans also face the challenge of osmotic diuresis; if urea in the collecting ducts is highly concentrated and UT regulation is lacking, it will draw water from the kidney interstitium and lead to undesirably increased water excretion. This means that UT is also essential to cetacean osmoregulation. Recently, a few studies have been conducted to investigate UTs in cetaceans. Janech and colleagues sequenced the first cetacean UT-A2 from the kidney of a short-finned pilot whale (whUT-A2)^45^. WhUT-A2 shows a high level of sequence identity to terrestrial mammals, and greatly enhanced the urea uptake of Xenopus oocytes in a heterologous expression experiment, indicating that the whale UT-A2 conducts urea. Subsequently, several UT-A2 genes from the kidney of baleen and toothed whales were cloned. It is proposed that there are possible regulation differences between these genes (different protein phosphorylation sites) leading to their high urinary concentration capability^46^; however, other studies indicate that UT-A2 is not stimulated by cAMP analog-dependent protein phosphorylation^28^,^47^,^48^,^49^,^50^. In 2009 and 2012, Levin and colleagues used X-ray crystallography to study urea transporter structures in detail, and they elucidated the permeation mechanisms of UT-B and dvUT in bovine and bacteria^51^,^52^. They indicated that both dvUT and UT-B are homotrimers and each subunit contains a urea conduction pore with a narrow selectivity filter. The binding sites in the filter and the well-placed α-helix dipoles accommodate multiple dehydrated urea molecules in single file that pass through the pore, acting like a molecular version of a coin-operated machine^29^. However, previous studies have not yet experimentally determined the structure and mechanisms of UT-A2, neither have they resolved the effects of molecular adaptation and physicochemical evolution of UTs. These pieces of information will be important for understanding the evolutionary urine-concentrating strategies adjustments that occurred in organisms to counteract external osmotic changes and providing a treasure trove of information on UT protein structure and function.

Marine cetaceans that inhabit a hyperosmotic environment (with little to no access to fresh water) appear to conserve water better and produce more highly concentrated urine (1353–2129 mOsm/kg) than humans and cattle, to which cetaceans are closely and can access fresh water from time to time^10^,^12^,^14^. Our recent studies showed that the Yangtze finless porpoise (*Neophocaena asiaeorientalis asiaeorientalis*, YFP), which resides in hypo-osmotic freshwater (Yangtze river) likely excretes relatively less concentrated urine (934 mOsm/kg) compared to its marine counterparts that include Bohai finless porpoises (*Neophocaena asiaeorientalis sunameri*)^25^. These observations regarding urine concentration lead to two questions. 1) Have the UT-A2 genes (which are correlated with urine concentration abilities in mammals) evolved adaptively during cladogenesis, when cetaceans transitioned from land to the marine environments? And 2) are there any molecular adaptations in UT-A2 between the extant seawater and freshwater cetaceans that could provide an adaptive advantage in facing different osmoregulatory challenges?

To address these questions, we sequenced the urea transporter UT-A2 from the kidney of freshwater YFP. Bioinformatics analyses of phylogenetic, molecular evolution and radical amino acid property changes were conducted on UT-A2s among cetaceans (both marine and freshwater cetaceans) and several terrestrial mammals to investigate the molecular evolution and adaptation of UT-A2s. Additionally, the newly revealed dvUT and UT-B 3D structure and mechanisms were introduced to explore the structural and functional conservation and variation of mammalian UT-A2. Furthermore, the molecular differences in the UT-A2 protein that may lead to function divergence between cetaceans and terrestrial mammals were discussed. These analyses may provide new insight in understanding the molecular mechanism of urea permeation and the molecular basis of osmoregulation in cetaceans and provide instructive information for understanding human kidney diseases correlated with urea regulation.

## Results

### UT-A2 sequences

**Figure 1:**
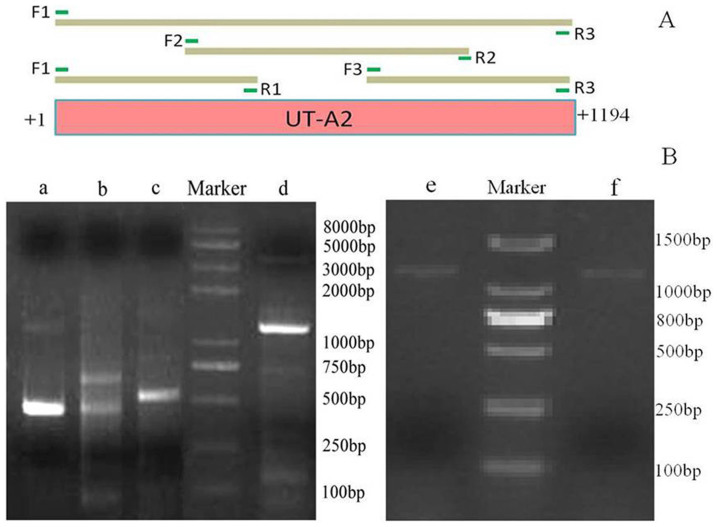
RT-PCR amplification of Yangtze finless porpoise UT-A2. (A) Schematic diagram of the UT-A2 coding sequence. The primers used for RT-PCR are indicated by the green bar and labeled with letters F and R. The amplified fragments for the following sequencing and splicing are indicated by the dark brown bars. (B) Products of RT-PCR amplification with a set of primers. Lane a: a 454-bp fragment amplified using the primers F1 and R1; lane b: a 652-bp fragment amplified using the primers F2 and R2; lane c: a 534-bp products amplified using the primers F3 and R3; lane d, e and f: the full length 1194-bp fragment amplified using the primers F1 and R3 from two Yangtze finless porpoise individuals.

As shown in Fig. 1B, the fragments and entire length of UT-A2 were amplified from mRNAs of two YFP specimens, using RT-PCR with the degenerate primers F1-R1, F2-R2, F3-R3 and F1-R3 (Table S1). After sequencing, splicing, and manually checking the amplicons, the full-length contiguous coding sequence of 1194 bp, encoding a 397-amino acid protein (the sequences of UT-A2 from the two YFP were absolutely concordant) was obtained. The alignments of coding sequences (CDS) and amino acids from UT-A2 of cetaceans and terrestrial mammals are shown in Fig. S1. The CDS of the YFP UT-A2 gene was submitted to the NCBI Data Bank (Gene Bank accession no.: KP318829).

### Phylogenetic analysis

**Figure 2:**
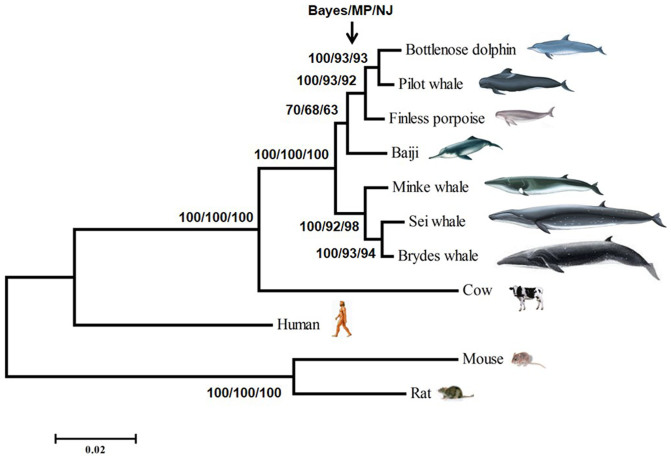
Phylogram resulting from NJ, MP and Bayesian analyses on UT-A2 genes from several cetaceans and terrestrial mammals. The numbers on each branch denote the bootstrap proportion inferred by each method. The cetacean drawings were modified from the book of Chinese cetaceans^107^.

In MrBayes analysis, the best-fit HKY + G model (−lnL = 3452.8572, K = 24, BIC = 7075.7559) was selected by Modeltest 3.7 using the Bayesian Information Criterion (BIC)^53^. The base frequencies of A, T, C, G were 0.2118, 0.3185, 0.2423 and 0.2275, respectively. The transition/transversion (Ti/Tv) ratio was 2.9435, and the putative variable site gamma distribution shape parameter was 0.2360. After the phylogenetic analyses, very similar dendrograms, with high bootstrap values, were constructed in all cases (Fig. 2).

### Molecular evolutionary analysis

**Figure 3:**
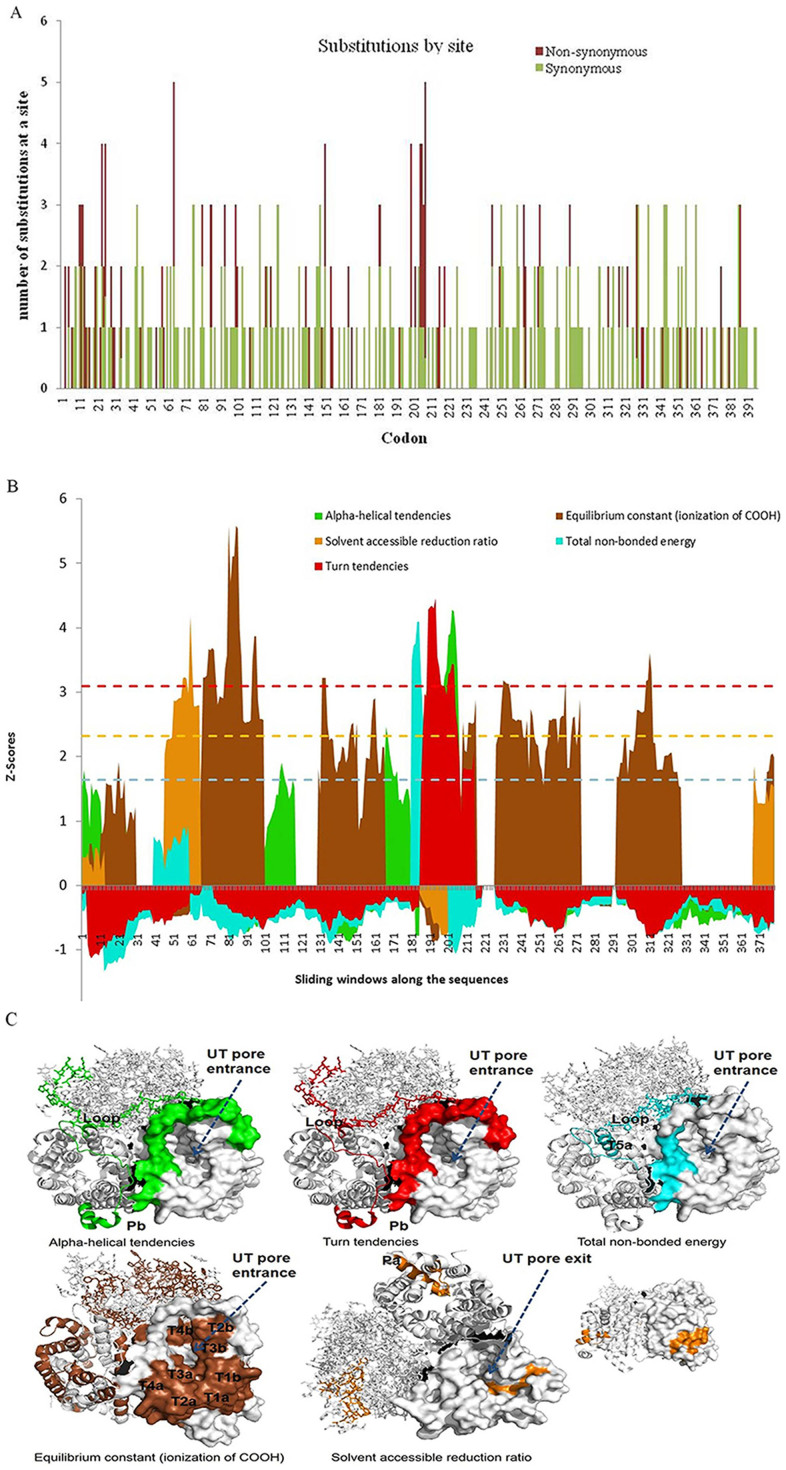
SLAC-inferred substitutions by site and detection of the positive destabilizing selection of the amino acids that were picked up by the sliding windows (windows size = 20) using TreeSAAP3.2. (A) The number of synonymous and non-synonymous substitutions on each codon were counted. For counting substitutions, the shortest evolutionary paths are assumed, and when multiples exist, the average over all is taken. Fractional counts are due to averaging over multiple pathways when multiple nucleotides are substituted along a single branch. (B) Regions above the z-score of 3.09 were significantly different than neutrality. Five types of significant physicochemical amino acids changes were identified and noted in different colors. (C) The clustered radical amino acid shift regions were correlated with aspects of protein 3D structure.

The average ω ratio for overall sequence pairs was 0.085, 0.114 and 0.086, as calculated from MEGA4, DataMonkey and PAML codeml (M0), respectively, indicating purifying selection on UT-A2. In PAML tests, the estimated parameters of different models and the LRT results are provided in Table 1. Rejection of the M0 model by the free-ratios model (p < 0.01) presented the variable ω values among branches. In a further analysis, an *a-priori* hypothesis was tested by specifying the cetacean-specific branch with a different ω rate; the one-ratio model was rejected by an unrestricted two-ratios model (p < 0.05), and the alternative hypothesis (estimated ω) eliminated the constrained null hypothesis (ω1 = 1) in the LRT tests (p < 0.001). The ω value estimated for cetaceans was 0.14, about twice the value estimated for non-cetacean mammals (ω = 0.07). We also tested the freshwater cetacean lineages for the baiji and YFP, but these were not significant relative to the null models. To evaluate ω ratio variation among codon positions, an LRT test based on site-specific models M3 (K = 3) versus M0 and M3 (K = 2) versus M0 was conducted. Both results (p < 0.001) provided evidence for the existence of rate-heterogeneous selection pressure at amino acid sites. In the analyses, M2a had the same likelihood values and identical parameter estimates as M1a, indicating that approximately 93.3% of the sites are under purifying selection (ω = 0.05). M7 is not rejected in favor of M8, suggesting an ‘‘L’’ shape of the beta distribution (0.198, 1.738). In LRT tests, the branch-site Model A performed significantly better than model M1a (p < 0.01), but was not significant against the null model A (p = 0.14) in the cetacean branch. However, the branch-site Model B was significantly better (p < 0.05) than model M3 (K = 2), and identified 3 positive sites, which is consistent with Model A-M1a. In the DataMonkey tests, the SLAC tests inferred substitutions by site using the shortest evolutionary paths (Fig. 3A), and the branch-site model MEME showed evidence that 2 sites are under episodic diversifying selection.

### Detection of positive selection at the amino acid level

**Figure 4:**
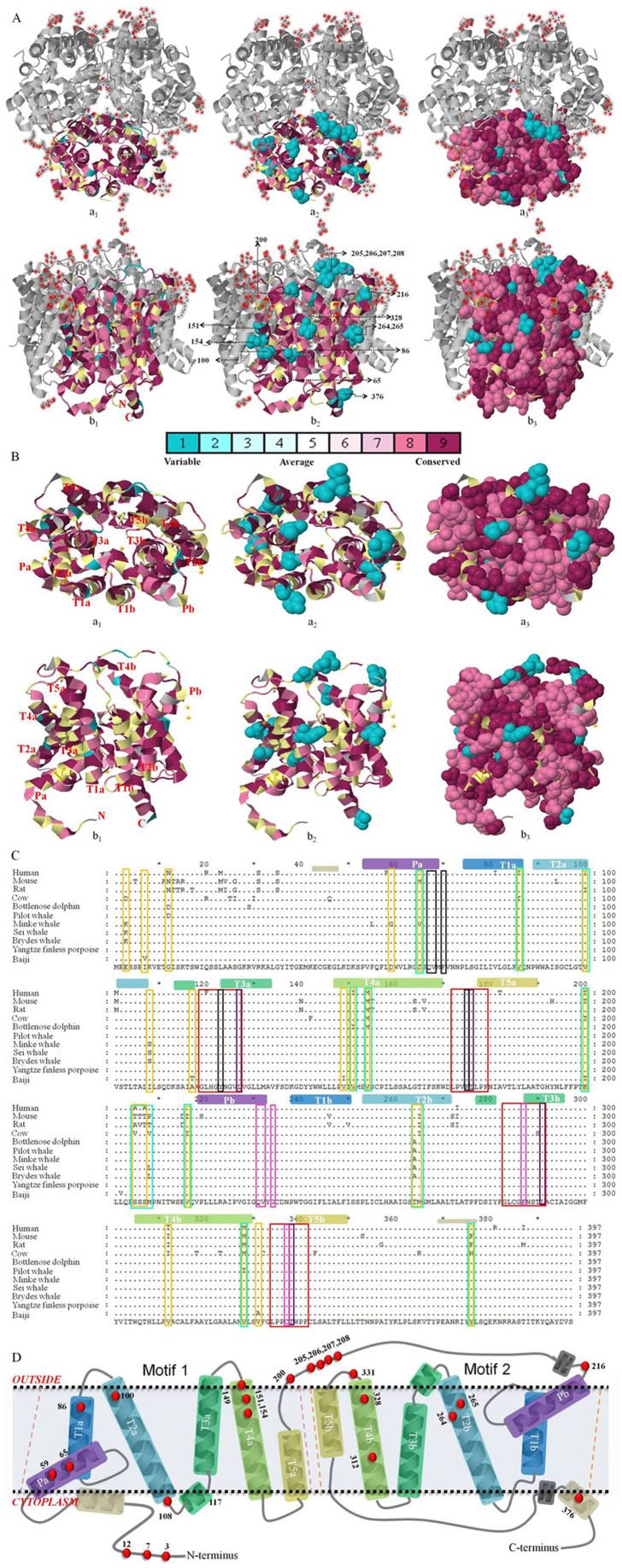
Topology and structure of the urea transporter superimposed with the multiple sequence alignment (MSA). (A) The *Bos taurus* UT-B trimer chain A was superimposed with MSA: *(a)* viewed from the extracellular side; *(b)* viewed from within the plane of the membrane. The amino acids of the monomer are colored by their conservation grades using the color-coding bar, with turquoise through maroon, indicating variable through conserved amino acid positions. *(a1)* cartoon representation of mapped UT; *(a2)* 15 variable residues were detected and presented using a space-filled model, the letter abbreviations ahead of the site-numbers are the corresponding *Bos taurus* UT-B amino acid, the letter abbreviations behind the site-numbers are the variable amino acids of the MSA; *(a3)* the variable and conserved sites were both presented using a space-filled model. (B) The superimposed dvUT viewed from the extracellular side (a) and from within the plane of the membrane (b). (C) The multiple amino acid sequence alignment of UT-A2. The colored bars indicated the location of intramembrane helices in the UT-A2 structure according to the superimposed dvUT and UT-B. The red-boxed residues correspond to the signature sequences of UT. The black-, petunia-, and pink-boxed residues correspond to the sites S_i_, S_m_, and S_o_, respectively, which line the selectivity filter of the UT. The cyan- and orange-boxed residues are the selected sites tested by Consurf and other evolutional assessment tools (treeSAAP, PAML and DataMonkey). (D) Annotated topology of the urea transporter with the evolutionary selective amino acid sites. The red dots represent the sites selected by PAML, DataMonkey, ConSurf and TreeSAAP.

**Figure 5:**
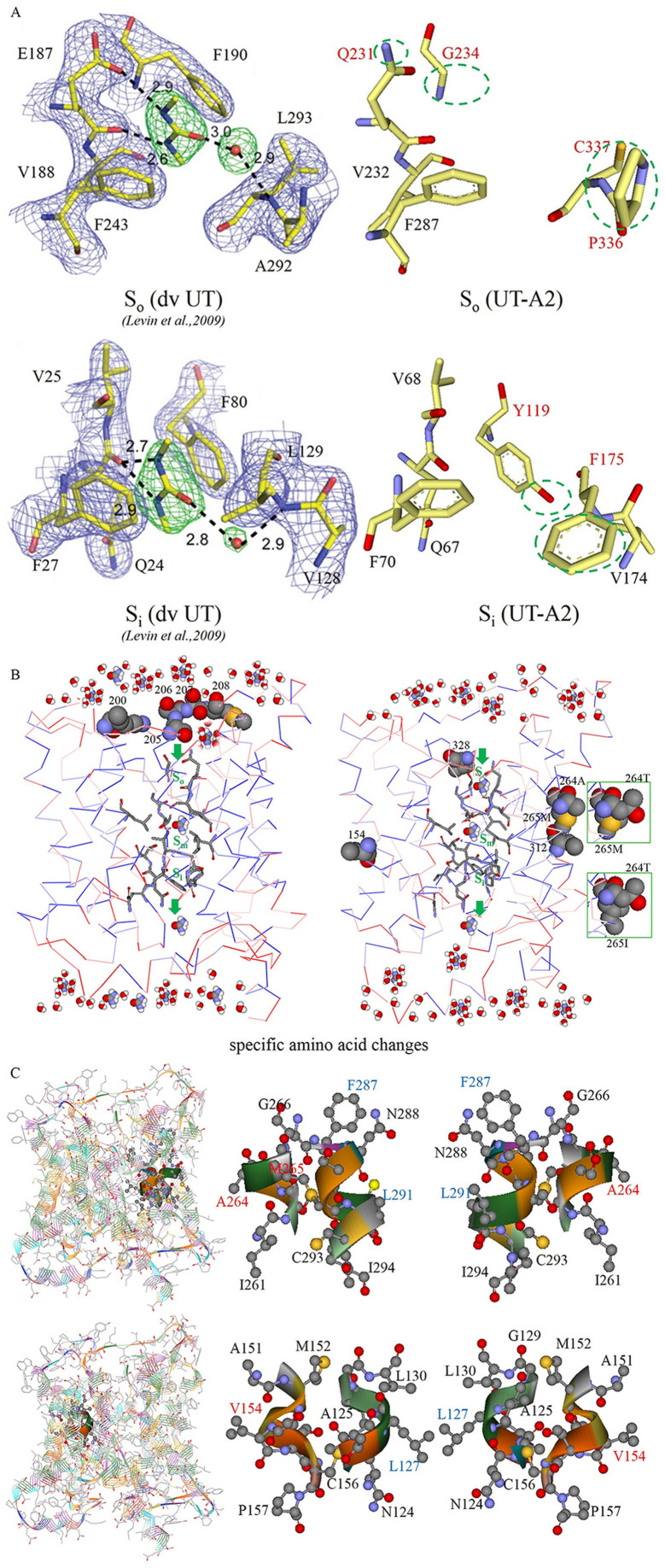
Specific amino acid changes of the UT-A2 protein. (A) Stereo view of residues in the selectivity filter S_o_ and S_i_ regions of UT-A2. (B) Specific amino acid changes among UT-A2s of the mammals. (C) Stereoviews of the mutation sites 264, 154 and the possible correlation sites.

To extrapolate the potential structure and mechanism of the UT-A2 protein, Consurf was introduced to compute the position-specific conservation scores. Then, graded conservation scores were projected onto the space-filled 3D structure of UT-A2's homolog dvUT and UT-B, and finally visualized (Fig. 4A, B): identical amino acids are shown in dark purple and variable ones in light blue. The results revealed that the functionally important regions on both the DNA and the protein were conserved. From the superimposed 3D structure, we can see that the individual protomers of UT-A2 have the same overall folding structure as do dvUT and UT-B, which contain two homologous halves with opposite orientations in the membrane (Fig. 4D, structural Motif 1 and Motif 2). A membrane-spanning pore is formed at the interface of the two halves that each contains six helices (Pa, T1a-5a and Pb, T1b-5b, Fig. 4B, C and D). For its important role in urea conduction, the differences between the UT selectivity filters in the mammalian UT-A2, UT-B and the bacterial dvUT were further investigated. The results indicated that the UT-A2 residues of the S_m_ regions (L127, T176, L291 and T338) were completely consistent with their corresponding sites in dvUT (L84, T130, L247, T294) and UT-B (L123, T172, L287, T334). However, the sites in the S_o_ and S_i_ regions showed some variation between dvUT, UT-B and UT-A2 (Fig. 5). The S_o_ region sites E187, F190, L293 of dvUT shifted to glutamine (Q231), glycine (G234) and cysteine (C337) in UT-A2, respectively (Fig. 5A). I228 in UT-B was replaced by valine in UT-A2 (V232), which is identical to V188 in dvUT. In the S_i_ region, F80 (G115 in UT-B) and L129 in dvUT shifted to tyrosine (Y123) and phenylalanine (F175) in UT-A2, respectively (Fig. 5 A).

Although these ligand binding sites in the selectivity filter changed among the UT-A2, dvUT and UT-B proteins, residues in the UT-A2 in all of these mammals were in full accord (Fig. 4C). To determine the molecular adaptations in UT-A2, TreeSAAP was further used for identifying and characterizing adaptation in terms of shifts in the physicochemical properties of amino acid replacements. Eight amino acid properties (alpha-helical tendencies (P_α_), bulkiness (B_l_), equilibrium constant-ionization of COOH (pK′), partial specific volume (V°), power to be at the C-terminus (α_c_), solvent accessible reduction ratio (R_α_), total non-bonded energy (E_t_) and turn tendency (P_t_)) were found to be undergoing positive-destabilizing selection (p < 0.001). To maximize accuracy, the three properties B_l_, V° and α_c_ that tend to generate false-positive results were eliminated from the results^54^. The sliding window graphs illustrate the spatial dynamics of selection across the primary sequence of the UT-A2 gene (Fig. 3B). Regions clustering around the radical physiochemical shifts (categories 6, 7 and 8, p < 0.001) from natural amino acid replacements were analyzed and visualized. The correlated 3D structure of the urea transporter showed the influenced protein regions that have been historically affected by selection on specific amino acid properties (Fig. 3C). The five amino acid property changes involve the protein secondary structure (P_α_ and P_t_), ionization constants (pK′), non-bonded energy (E_t_) and solvent accessibility (R_α_). P_α_ and P_t_ are prone to be clustered at the apices of T5a, Pb and the loop between these helices. E_t_ tends to be clustered at the apices of T5a and the loop affecting the non-bonded energies. Both of these radical changes are in the entrance region of the urea conduit. The shifts in pK′ clustered at the surface of the protein and the entrance of the UT pore, whereas R_α_ is located at the exit region of the UT pore.

Among the non-synonymous substitutions, codons under positive-destabilizing selection were identified from 20 amino acid properties (the 11 exchanges that performed the worst were eliminated) in TreeSAAP and were summarized in Table 2. The major physicochemical amino acid properties that yielded positive analytical results are summarized in Table 3. The cetacean clade was detected by treeSAAP to have substitutions on sites 200, 205 and 312. Site 200 is located on the loop between T5a and Pb. The substitution (Thr → Lys) on this site could increase the protein pH_i_ and decrease the E_t_ property. The amino acid shift from Ala to Ser at site 205 and the substitutions at sites 206 and 207 are located on the apical loop of the periplasmic side between T5a and Pb, which could influence the conformation of the protein (decrease P_α_ and increase P_c_ and P_t_). The substitution at site 312 (Ile → Val), which is located on the T4b helix cytoplasmic side, could increase the pK′. Baleen whales underwent positive-destabilizing selection at sites 3 and 108. Site 3 in the N-terminus (Glu → Lys); pHi was increased, whereas site 108 (Ile → Ser), which is located on the cytoplasm side of T2a helix, could cause many radical amino acid property changes (N_s_, B_r_, R_F_, P_c_, pK′, F, R_α_, H_t_ and P_t_). We found that freshwater YFP and baiji shared two identical amino acids at sites 264 and 12, whereas marine toothed whales have substitutions of Thr to Ala and Gly to Asp. These changes could increase the P_α_ of T2b and decrease the E_t_ of the N-terminus. There are some substitutions pertaining to baiji (sites 7, 149 and 200) and minke whale (sites 59 and 65), which are different from their counterparts. The properties of the amino acid at site 200 in the baiji displayed a decrease in pH_i_ and an increase in E_t_. Substitution at site 7 (Ile → Val), which is in the N-terminal region, could increase the pK′, whereas substitution at site 149 (Val → Ile), which is in the T4a helix on the periplasmic side, could also decrease the pK′. In this study, we also detected some selection sites pertaining to baleen whale UT-A2 (sites 3 and 108). At site 59, substitution (Asp → Gly) increased the E_t_. At site 65, substitution (Thr → Val) increased the N_s_ and R_α_. The radical amino acid changes were marked on the multiple sequences alignment (MSA) (Fig. 4C), the topology (Fig. 4D) and the 3D structure of the urea transporter (Fig. 5B).

## Discussion

The UT is a type of integral membrane protein that facilitates the diffusion of the osmolytic urea and plays important roles in *in vivo* water equilibrium regulation^26^,^28^,^35^,^51^,^52^,^55^,^56^. In the present study, we looked for evidence of adaptive molecular evolution of the osmoregulation-correlated gene UT-A2 in the cetacean lineage (marine and freshwater), relative to their terrestrial counterparts. We cloned the UT-A2 gene from kidneys of two freshwater YFP and bioinformatics analyses were performed. The gene was found to be highly conserved (87–99% identity) and undergoing purifying selection. However, the cetacean branch has a significantly higher ω ratio relative to terrestrial mammals. Different selection sites in the cetacean branches were picked up with several complementary approaches, including d_N_/d_S_ (PAML, Datamonkey), MM01 (TreeSAAP) and Rate4site (ConSurf) (Table 2). Furthermore, we found that the UT-A2 individual protomer has an overall fold similarity with dvUT and UT-B (Fig. 4), although several variants occurred in the functional S_o_ and S_i_ regions of the UT selectivity filter (Fig. 5A). Finally, the radical amino acid changes (particular substitutions and cluster regions properties) were superimposed on the 3D structure (Fig. 3C and 5B, C), and then the molecular adaptations that may lead to the potential functional differences were discussed based on the urea permeation mechanisms of dvUT, UT-B and the urea channel of *Helicobacter pylori*^51^,^52^,^57^.

In the evolutionary process, molecular adaptations that optimize the organism's survival in a given habitat can be accumulated and inherited. Effectively neutral nucleotide substitutions, and either purifying selection (maintaining functions) or positive selection (favoring new functions), play major roles in evolution at the molecular level. To understand what evolutionary forces drive protein evolution and why protein sequences differ in different species, several methods have been proposed to detect selection or adaptation since the genetic code was discovered in the 1960s. The most common or broadly accepted approach is the family of methods that contrast the rates of nonsynonymous and synonymous substitutions (d_N_/d_S_, also known as Ka/Ks or ω); these methods have enjoyed a great deal of success^58^,^59^. However, because most substitutions are expected to be neutral or deleterious^60^, adaptive changes will be difficult to find using ω ratios, particularly if we only look for ratios greater than one^61^. In fact, relatively few mean ω estimates of genes in cetaceans are significantly greater than 1 (e.g., only 228/10,000 were found in a bottlenose dolphin and nine other genome comparisons^62^ and only 24/10,423 were found in the common branch of the baiji and dolphin versus the other four genomes^63^). Additionally, for a conserved gene, sometimes even a single amino acid change can be adaptive if it provides a biochemically advantage relative to contemporaneous alternatives, which seems not sensitive enough for the d_N_/d_S_ method^64^,^65^,^66^,^67^,^68^. In the analyses of nucleotide substitution patterns in this study, the ω ratios tested as significantly less than 1 (approximately 0.1), showing that a strong purifying selection plays a central role in the evolution of UT to keep its important functions in urea conduction. Furthermore, to find the drivers of adaptation to the different levels of osmoregulatory challenges (different urea levels generated by protein catabolism and infrequent access to water), branch-specific, site and branch-site models were conducted to explore the variation of evolution rates in specific sites in cetacean-specific, baiji and YFP lineages. However, the results showed weak evidence of positive selection using this pattern (Table 1). The two-ratio model detected that the baiji and cetacean-specific branches have a significantly higher evolutionary rate than the background, indicating that the constraint on functional change of UT-A2 in the cetacean and baiji branches has been relaxed. Although LRT tests of Model B-M3 (K = 2) (p < 0.05) or MA-M1a (p < 0.01) picked up several positively selected sites in the cetacean and baiji clades, the result of the stringent testing (*test 2*, MA *vs* MA null) is not significant^69^.

As a reaction to potential bias in d_N_/d_S_ patterns^64^,^66^,^67^,^68^, we applied a physicochemical property model (MM01 methods) to the possible molecular adaptation of UT-A2 among cetaceans, which has been proven to be a sensitive way to detect the subtle effects of positive selection^64^,^66^,^68^,^70^. Because the 3D structure of a protein determines its function, the molecular adaptations resulting from changes in physicochemical amino acid properties were discussed based on the revealed 3D structure and mechanisms of the UT analogs. In the analysis of this pattern, five radical amino acid properties changes that involve protein secondary structure (P_α_ and P_t_), ionization constants (pK′), non-bonded energy (E_t_) and solvent accessibility (R_α_) were detected using the sliding window mode (Fig. 3B, C). Using the analytical software TreeSAAP, we also characterized several positive destabilizing selections (selection that results in radical structural or functional shifts in local regions of the protein) that correlated with 12 biochemical shifts (Table 2), which represented the unambiguous signature of molecular adaptation^64^.

In the 3D structure characterization, we found that the individual protomer of UT-A2 has the same overall folding pattern as do dvUT and UT-B (Fig. 4), containing 6 pairs of well-placed α-helices, which form the urea conduction pore. The conserved amino acids of the S_o_ region (Q231, V232, G234 and C337), S_m_ region (L127, T176, L291 and T338) and S_i_ region (V68, Q 67, Y123 and F175) are brought together to form the selectivity filter that helps urea to travel through the protein; these are stick-presented in Fig. 5B. When we compared the UT-A2 selectivity filter with that of dvUT, several residue changes in the S_o_ and S_i_ regions (Fig. 5A) were observed, indicating the potential differences in urea permeation regulation of these two proteins. The shift from F (phenylalanine) to G (glycine) on site 234, minus the planar, aromatic side chains, which were theorized to fix the orientation of the urea molecules entering the pore^51^, as in coin access to “coin slots”^29^, may indicate a structural relaxation of the UT-A2 entrance and supply enough space for urea passing through when there is a higher urea concentration in the periplasm. The shift from E (glutamic acid) to Q (glutamine) on site 231, along with a change from the carbonyl to amide group, which retains significant polarity and is a rich source of hydrogen bonds, would make it easier to bind urea and urea-water compounds. These two site shifts may functionally increase the rate and efficiency of urea conduction through enlargement of the urea acceptability of the entrance. The hypothesis could be verified by structural and functional studies of UT-B^51^, which displays nearly the same amino acids shifts. In the S_i_ region, F80 and L129 shifted to Y119 and F175, respectively, adding a hydroxyl and phenyl group. The hydroxyl of Y119 could be a binding site for urea and the hydrophobic phenyl group likely helps the coin-shaped urea molecule to exit in good order, which may be important for osmotic changes in the cytoplasm. These two sites likely also increase the efficiency of urea discharge. To conclude, compared to bacterial dvUT, the urea conduction pore of UT-A2 seems to have a structurally relaxed entrance and a much more orderly exit, as well as enhanced urea binding capacity; therefore, it could promote the rate of urea conduction, which might be a demand caused by the higher efficiency of protein catabolism in mammals.

After superimposing the molecular adaptation sites onto the topology and 3D structure (Fig. 4, 5) we found that the positive-destabilizing selection in cetaceans mainly occurred at the N-terminus, the periplasmic side of the helices and loops, and the outside helices (T2a, b and T4a, b) of the UT-A2 protein (Table 2). We found that most of the radical amino acid changes are located on the regions that were exposed to the solvent, versus those buried in the interior of the folded structure. This may indicate that the interior residues are subject to stronger constraints against the exposed residues, which is consistent with the sliding-window amino acid properties cluster analyses (Fig. 3C). Conant suggested that the phenomenon of “neutral evolution on mammalian protein surfaces” was due to the selection to *Ne* (the variation in effective population sizes)^71^. However, TreeSAAP, the method that we used for detecting the amino acid changes, is not based on mutation frequencies within populations, but upon the sequences themselves^72^. Therefore, in this study, we assume that a few residues (e.g., the interacting surface) were under selective constraint or directional selection. The sliding-window analyses indicated that the entrance region of UT-A2 has undergone P_α_, E_t_ and P_t_ property changes (Fig. 3C), which may influence the accessibility of protein interfaces and interactions with other molecules^73^,^74^. Previous studies suggested that residues in the entrance region of urea channel are responsible for gathering urea-water compounds and gating urea entrance through the pore^75^. In the absence of an experimental loop structure of a urea transporter or urea channel, the function of the loop was not yet well illustrated^51^,^52^,^75^. Mutation studies of the *Helicobacter pylori* urea channel have shown that the histidine residues on the periplasmic loops are essential for pH gating^76^, suggesting that the opening and closing of the channel might be controlled by reversible folding of one or both loops into the periplasmic vestibule of the channel^75^. In this study, several radical amino acid substitutions occurred on sites 200, 205, 206 and 207, which are located on the periplasmic loop (Fig. 5B). Residue 200 (Thr → Lys, hydroxyl amino acid, lower side chain flexibility and polar to methylene groups crowned with a amine nitrogen, higher side chain flexibility, ionic interaction mode, lower hydrophobicity and positive property^77^) could increase the protein pH_i_ (Tables 2, 3) and make the isoelectric point more basic, decreasing its tendency to aggregate at or below neutral pH due to electrostatic interactions^78^. This may have a role similar to histidine residues on the loops of the *Helicobacter pylori* urea channel, affecting the gating of the UT-A2 protein. Furthermore, the codon substitution decreases the E_t_ property, affecting the short-, medium- and long-range interactions with atoms in the same and other molecules^73^,^74^. Site 205 (Ala → Ser, to 3 potential side chain H-bonds and polar) is also located on the apical loop of the periplasm side between T5a and Pb, which could decrease P_α_ and increase P_c_ and P_t_ (Tables 2, 3), resulting in increased accessibility at protein interfaces^73^,^79^. Together with the Ser at site 206, 207, it provides a relatively larger polar surface binding the water-urea compounds^80^, and could potentially gather more urea to enter the urea conduction pore. Site 328 (Met → Val, shift to lower side chain flexibility and slight higher hydrophobicity) is also located on the region adjacent to the entrance to the pore (Fig. 5B). Met and Val have different restrictive capacities (ΔS^contact^) from other hydrophobic residues, which could affect the conformation and function of a protein^81^. In the α-helix, aliphatic residues (Leu, Val, Ile) more strongly affect each other, whereas Met residues are less affected by other residues^81^. Therefore, the apical side of the T4b helix of cetaceans seems to be more restricted, and may help to provide a wider entrance space for the urea transporter, affecting the protein functional activity. In previous studies, shifts in the Met and Val residues have been found to affect some genes' activity and function^82^,^83^. Other types of amino acid changes in the cetacean occurred on the surface of UT-A2, such as sites 154 and 264. Site 154 also had a shift of Met to Val in the cetacean branch, along with a decrease of side chain flexibility and an increase in hydrophobicity^77^, which has different restrictive capacities (ΔS^contact^) and could affect the conformation and function of a protein^81^. When we mapped the site on the 3D structure of UT-A2, we found that the region, where the site occupied, was across from the S_m_ region of the urea conduction pore (Fig. 5C), which may indirectly affect the urea conduction through the changes in ΔS^contact^ or through conformation changes. Site 264, which is located on the T2b helix, has an amino acid shift of Thr to Ala (change to limited side chain flexibility, no H-bonds, higher hydrophobicity and nonpolar^77^) in cetaceans except for the freshwater YFP and baiji. These changes could increase the P_α_ of T2b (Table 2), leading to a longer, more rigid alpha helix with a more stable lipid raft composition, which, in turn, may alter its interactions with amino acid motifs and its efficiency of combination with other substances^84^,^85^; thus, it may raise the efficiency of UT-A2 of marine cetaceans compared with the freshwater cetaceans. The region was across from the S_m_ region of the urea selectivity filter regulating the rate of urea conduction, which may increase the hydrophobicity of the S_m_ region, and raise the energy of dehydration, affecting urea conduction (Fig. 5C). The third type of amino acid changes occurred on the N-terminus of UT-A2. On site 3 (Glu → Lys, containing a carboxylic acid group and negative charge to containing amine nitrogen and positive charge^77^), the pH_i_ was increased, thus altering the interaction with electrostatic particles and the surrounding residues or subunits^73^,^78^ (Tables 2, 3). On site 12, marine toothed whales have the substitution of Gly to Asp (a change to moderate side chain flexibility, ionic interaction mode, four potential side chain H-bonds, lower hydrophobicity and polar properties^77^), which can decrease the E_t_, affecting the interaction of the amino acid with the surrounding atoms, residues, or subunits^73^,^86^. However, in the absence of functional studies of the N-terminus of the urea transporter or urea channel, we cannot deduce the role of amino acid changes within this region. Only when mutagenesis studies have been accomplished can this issue be illustrated. However, in this study, we predict some potential mutations that may influence protein function, which may help narrow down where scientists should concentrate future research. Collectively, we found conservation of the urea selectivity filter of UT-A2, despite some changes in the S_o_ and S_i_ regions, versus bacterial dvUT, which may correlate with the adaptation of high-level protein catabolism in mammals. Also the radical amino acid changes tend to occur on the surface of the protein and may generate mildly advantageous effects for adapting to a changed environment. The selection may provide cetaceans a relaxed or wider urea channel entrance or increased S_m_ hydrophobicity to enhance the efficiency of urea conduction. However, in the absence of mutagenesis experiments, the conclusions drawn from the simulations remain speculative.

Based on these selection-pattern analyses and the potential functional characterization, we suspect that the period of adaptive evolution and rapid amino acid substitution may only have occurred for a short time period, followed by a long period of selective constraint (purifying selection) on the improved protein, which would obscure the selective processes and make it difficult to detect positive selection using the d_N_/d_S_ pattern^87^. The similar evolutionary rates in the branches of freshwater and marine whales suggest that baiji and YFPs may have similar molecular UT adaptations to their marine counterparts, although they live in freshwater and generate relatively diluted urine^25^. In fact, the higher level urea transporting capacity that they inherited from their marine ancestor is not a burden to their physiological functions, as they can easily obtain sufficient fresh water from the environment to dilute the urine and eliminate the redundant urea. Physical and biochemical analyses of plasma and urine showed that both baiji and YFP excrete urine and that the urea concentration in the animals is still higher than the concentration in plasma^25^,^88^, indicating active transport of urea in the kidney. The physical experiment (fresh and seawater ingestion) on bottlenose dolphins has shown that if a marine toothed whale were provided with sufficient freshwater, it would also excrete relatively diluted urine^12^. When a certain amount of deionized water was fed to the bottlenose dolphin, the urine osmolality could decrease to 813 ± 30 mOsm/kg, and the urea concentration in urine could decrease to 11–38 mEq/L, similar levels to those in YFP and baiji^12^,^25^,^88^. The real challenge that the baiji and YFP face may be to produce more diluted urine (e.g. adaptation of water regulation through aquaporin). We believe that this hypothesis can be further confirmed, as UT-A2 sequences from marine finless porpoises are sequenced and the water channels are studied in detail. The other question is: why is the UT-A2 of YFP much more conserved with marine toothed whales than baiji, although baiji and YFP reside in similarly adapted osmotic conditions (similar osmosis, food and similar freshwater surroundings)? We attribute the dissimilarity to relaxation of purifying selection of UT-A2 in baiji. There are two aspects to purifying selection: (1) past selection works against highly deleterious mutations, which are eliminated quickly; and (2) ongoing selection works against slightly deleterious mutations, which may be relatively inefficient if the effective population size is small^89^,^90^. Previous studies suggest that river dolphins, including baiji, escaped extinction and adapted to their riverine habitats during continental flooding in the Middle Miocene caused by the rise in global sea levels^91^,^92^,^93^. Thus, we argue that baiji must have undergone reduced effective population sizes (*N_e_*) that were influenced by oceanic events in their long-term fresh-water living conditions (approximately 21.5 Ma)^92^, and have experienced less efficient purifying selection. They are expected to accumulate more slightly deleterious mutations and thus to show a greater dissimilarity to their ancestors. The genome study of baiji also suggested that a bottleneck occurred near the end of the last deglaciation^63^, which is consistent with our hypothesis. From another viewpoint, a change in effective population size can be distinguished from a change in selection intensity because a change in population size is expected to affect all genes along a lineage, producing a genome-wide effect on the ω ratio^94^,^95^. In this study, a variance no greater than expected was observed between the d_N_/d_S_ of the UT-A2 of the baiji lineage (0.39) and the baiji genome d_N_/d_S_ (0.32)^63^, also indicating that the relatively increased ω value was due to the confounding effects of population demographic history. However, YFP, the sole species of freshwater finless porpoise, entered the Yangtze River approximately 2.00–0.72 Ma along with the Quaternary glaciations^96^,^97^,^98^, and are considered to have diverged from their marine origin in the Yellow Sea. Until now, YFP was classified as a narrow-ridged finless porpoise species (*N. asiaeorientalis*), with the other marine subspecies of East Asian finless porpoise (*N. a. sunameri*)^99^,^100^. If that were the case, it means that the subdivision and freshwater adaptation of the YFP was a recent historical event. Therefore the baiji have likely lived for a much longer period of time in fresh water and may have undergone slightly deleterious mutations that are reflected in their differential base substitutions, and even in the molecular divergence of their UT-A2.

It happens that there is a similar case, marine osmosis habitats, where the driving forces of UT-A2 are different between the baleen whale and toothed whale. One hypothesis is that differential feeding led to different urea concentration levels of their internal milieu along with different selective pressures on the UT gene. Marine toothed whales such as bottlenose dolphins and pilot whales feed mainly on marine fish, whereas baleen whales, such as Bryde's whale and sei whales, feed on invertebrates^20^. Most invertebrates are osmoconformers, such that whales ingest seawater passively. Consequently, through food ingestion, relatively higher levels of urea concentration could be observed in the internal milieu of toothed whales^10^,^14^,^25^. The physiological and biochemical analyses of cetacean plasma and urine also indicate the importance of feeding for osmoregulation^14^. This hypothesis is limited to explaining the different relaxed selection constraints that occurred on UT-A2s between minke whale and the sei/Bryde's whale, and between the baiji lineage and YFP lineage, as they feed on nearly the same osmotic foods and reside in the same aquatic surroundings^20^. In this instance, a new or modified function is not necessary for the already adapted gene. The MEME results further indicate that some sites of marine toothed whale (site 12, empirical Bayes factor > 100) and minke whale (site 65, empirical Bayes factor > 1000) are under episodic diversification, indicating that the selective forces on UT-A2 are not constant across taxa. Therefore, there could be some transient periods of adaptive evolution masked by the prevalence of purifying or neutral selection on other branches. We argue that UT-A2 of marine cetaceans must have undergone different transitional periods of adaptation, influenced by these major oceanographic events, and that they are expected to have accumulated different mutations and thus to show a greater dissimilarity. In terms of potential positive selection, relaxation of purifying selection and episodic diversification in independent lineages of cetaceans in similar environments, we conclude that UT-A2s might have been periodically adjusted during or after cladogenesis and undergone periods of increased standing genetic variation, including mutations of small-to-modest effect that would allow adaptation to changing osmotic environments.

In summary, we found that a strong purifying selection plays a central role in the evolution of the urea transporter to maintain its important urea conduction function. We suggest that positive-destabilizing selection, relaxation of purifying selection and episodic diversification and play important roles in the process of cetaceans reinvading the sea and subsequently transitioning to different osmotic environments; this means that UT-A2 experienced standing genetic variation or mutations and has periodically adjusted during or after cladogenesis. We suggest that the freshwater baiji and YFP have basically retained the molecular adaptations of their marine counterparts, although two common amino acid sites (sites 264 and 12) along with shifts of the properties P_α_ and E_t_ that differ from their homologous marine toothed whales were detected. We found that the UT-A2 protein has folding similarity with dvUT and UT-B, whereas some variants occurred in the functional S_o_ and S_i_ regions of the selectivity filter. We also contribute to the knowledge of physiochemical and molecular adaptive evolution of UT-A2 by identifying several amino acid property shifts (pK′, P_α_, PH_i_, E_t_, *et al*.) that may generate mildly advantageous adaptations, which occurred at the entrance and surface of the UT-A2 protein. The conservation of amino acid residues within the selectivity filter of the urea conduction pore is likely necessary for urea conduction, whereas the radical amino acid replacements around the entrance and exit of the conduction pore could potentially affect the activity of the UT-A2 protein. Collectively, this study identified regions in UT proteins that are not as highly conserved as the selectivity filter region, but may play important roles in the activity of the UT protein. Further studies are necessary, however, to identify the function of N-terminus and to determine the effects of the radical amino acid changes.

## Methods

### Tissue collection

The kidney samples used for this study were collected from two freshly dead YFP (within 3 hours of death). One accidentally injured female YFP, with a body length of 128 cm, was found in the Shishou stretch of the Yangtze River; the other YFP, a male with a body length of 95 cm, was by-caught by a fisherman in the Honghu stretch. All possible rescue efforts had been strictly conducted following the Regulations of the People's Republic of China for the Implementation of Wild Aquatic Animal Protection (promulgated in 1993). Necropsy and sampling were conducted systematically in accordance with all ethical guidelines and legal requirements in China. The protocol of this study was approved by the Institutional Review Board of the Institute of Hydrobiology, Chinese Academy of Sciences.

### Amplification and sequencing

Total RNA was extracted using TRIzol (Invitrogen), and then run on a 1.0% agarose gel to confirm its integrity and purity. First-strand cDNA was synthesized from the total RNA using a First Strand cDNA Synthesis Kit (Fermentas). The cDNA served as a template for PCR amplification, using the specific primers in Table S1 (the red bars in the schematic diagram Fig. 1A), which were designed on the basis of the sequences of UT-A2 cDNAs of several cetaceans and terrestrial mammals that were previously reported^45^,^46^. The PCR amplification reaction conditions were as follows: initial denaturation at 94°C for 3 min, followed by 29 cycles of 94°C for 30 sec, 53°C for 30 sec, and 72°C for 75 sec, and ending with an additional 7 min at 72°C. Amplified PCR products were purified after running on an agarose gel and then cloned into a topTA2 vector (Toyobo). After extracting the plasmid DNA, nucleotide sequencing was conducted by Shanghai Majorbio Bio-Pharm Technology Company. Sequence data were assembled using the DNA Star Seq Man program and manually checked for accuracy.

### Database searches for UT-A2 genes

We collected 10 publicly available UT-A2 coding sequences from the DDBJ (http://www.ddbj.nig.ac.jp/), NCBI (http://www.ncbi.nlm.nih.gov/), and Ensembl (http://www.ensembl.org/index.html) databases (Date of access: 01/09/2013) for analyses, including five marine cetaceans (*Globicephala macrorhynchus*, GenBank Accession No. AY061881; *Balaenoptera acutorostrata*, AB266067; *Balaenoptera borealis*, AB266069; *Balaenoptera brydei*, AB266070; *Tursiops truncatus*, ENSTTRT00000007415), four terrestrial mammals (*Bos taurus*, ENSBTAT00000002712; *Homo sapiens*, NM_007baij163; *Rattus norvegicus*, U09957; *Mus musculus*, AF367359) and one river dolphin (*Lipotes vexillifer*, AUPI01117101.1)^63^.

### Phylogenetic Reconstruction

Nucleotide coding sequences and amino acid sequences of UT-A2 were aligned using CLUSTAL X (Thompson, et al. 1997) and the BioEdit program (Hall, 1999) aided by manual checking. An 1194 bp alignment of 10 UT-A2 coding sequences was used for phylogenetic analyses. Alignments were unambiguous because these UT-A2 sequences contained no obvious insertions or deletions. Modeltest 3.7 (Posada and Crandall, 1998) and MrMTgui (Nuin, 2007) were introduced for the selection of the best-fitting evolution model and the Bayesian Information Criterion (BIC) was preferred as a model decision criterion. Then, phylogenetic tree reconstructions were performed using MEGA4 for MP and NJ analyses (Tamura, et al. 2007), and MrBayes-3.1.2 for Bayesian estimation (Ronquist and Huelsenbeck 2003). Both MP and NJ trees were evaluated using bootstrap testing with 1000 replicates.

### Molecular evolutionary analysis

To investigate the possible role of positive selection on the evolution of UT-A2 gene in the cetacean clade, codeml (PAML) and DataMonkey were introduced. The codon substitution models were implemented in the PAML program with estimated transition/transversion rates and F3 × 4codon frequencies algorithm (Yang, 2007). A likelihood ratio test (LRT) was used to compare the fit of null models and alternative models, and the significance of the LRT statistic was determined by using a χ^2^ distribution. We applied various models, including branch-specific models, site-specific models and branch-site models to test the potential selection pressure acting on the UT-A2 gene. In these tests, the UT-A2 gene tree (Fig. 2) was introduced as the guide tree. In branch model testing, the LRT of the free-ratio model (an independent ω value for each branch) against the one-ratio model (a constant ω value across all branches) was performed to ensure variations in ω values among the branches. The two-ratio models were finally applied to test an *a-priori* hypothesis and confirm selection pressure on cetacean-specific, baiji and YFP branches by setting a foreground ratio ω_1_ while all other branches have the background ratio ω_0_, respectively. Also, the results were contrasted against a model of neutrality (fixed ω to 1). In the site model testing, M3 (discrete, fits k = 2 or k = 3 ω classes estimated from the data) against M0 (one average ω across all sites and all branches) was used to test for rate heterogeneity among amino acid sites; M7 (beta, which assumed a beta distribution of ω between 0 and 1) versus M8 (beta & ω, which added the extra ω > 1) and M1a (neutral, assumes two sites classes 0 < ω < 1 and ω = 1) versus M2a (selection, adds an additional site class of ω > 1) were used to identify the possible sites under selection. In the branch-site model testing, the cetacean-specific branch, baiji and YFP were defined as the foreground while all the other branches were defined as the background, and then test 1 (modified model A versus site model M1a), test 2 (modified model A versus branch-site model A with ω_2_ = 1 fixed) and model B (ω_0_ and ω_1_ are estimated as free parameters) versus M3 (with two discrete classes of sites) were conducted to detect sites under positive selection along cetacean lineages. In the positive-selection site and branch-site model tests, the conservative Bayes Empirical Bayes (BEB) analyses were also implemented to test the possible selection sites and their posterior probabilities^101^. To compensate for local optima effects in the analyses, the program ran two times, starting with ω values both above and below one. The web interface DataMonkey (Pond and Frost, Datamonkey, http://www.DataMonkey.org, 2005. Date of access: 26/09/2013), which uses the HyPhy package as its processing engine, was implemented as a complementary approach to identify the codons and lineages under selection. First, the best-fitting nucleotide model (HKY85, AIC = 7058.196, p = 0.003) was automatically selected. SLAC (single-likelihood ancestor counting) analyses^102^ was used to estimate the number of synonymous and non-synonymous substitutions of each codon along the UT-A2 sequences. Exploratory analysis was also conducted by MEME^103^ to assume that ω is not necessarily constant over time. To decrease the effect that purifying selection in some lineages might have to mask the signal of positive selection in others, and to measure the probability of observing a large d_N_/d_S_ in random neutral data, high nominal α-levels were advised^102^. Here, the default cut-off value of P < 0.1 was chosen.

### Amino acid properties and evolutionary conservation analyses

To detect significant physicochemical amino acid changes among the residues in the UT-A2 gene, selection tests were performed on the alignments and phylogenetic tree using TreeSAAP 3.2 (Woolley, et al. 2003), which is based on the MM01 mathematical model along with implemented modifications^54^,^64^,^70^. In the program testing, universal genetic codes, the HKY85 evolution model and 31 quantitative biochemical properties were used to estimate adaptive selection (referred to as positive destabilizing selection, when the frequency of changes in magnitude categories 6, 7 and 8 were highly significant, z-scores > 3.09 and P < 0.001). Changes in magnitude (categories 6 to 8) that were also significant (p < 0.05) were first identified with an overall analysis of our data. Evaluation of several sliding window sizes showed that a window of 20 amino acids (incremented every single site) provided the best signal-to-noise ratio, and its statistical significance was determined using a Bonferroni correction (α = 0.001; 99.9% confidence). The results of sliding window analyses were used to identify positive destabilizing selection regions for each property at a significance level of p < 0.001 (Z-Scores > 3.09)^54^,^64^,^66^. To improve detection accuracy, the amino acid properties that tend to generate false positives were eliminated, and only those that had an accuracy of detection of selection higher than 85% were considered^54^.

It is well known that the three-dimensional (3D) structure of a protein determines its function. To understand the potential functional adaptation of the UT-A2 protein, we further investigated the evolutionary conserved and variable positions, as well as their physicochemical shifts, based on the known 3D crystal structures of UT. According to Levin and colleagues^51^,^52^, the channel is lined by the residues from the highly conserved urea signature sequences^104^ at the interface of the two halves (Pa,T1a ~ T5a and Pb, T1b ~T5b) (Fig. 4C). The channel contains a constricted selectivity filter that can accommodate urea molecules in single file and be divided into three regions: S_o_, S_i_ and S_m_^51^,^52^. Given the high conservation of the “urea channel trimer” structure across the UT family^51^,^52^, we superimposed the evolutionary conservation scores onto the 3D structures, *Bos taurus* UT-B (PDB ID: 4EZC)^52^ and *Desulfovibrio vulgaris* dvUT (PDB ID: 3K3F)^51^, to examine evolutionarily conserved and variable regions among these homologous sequences of this protein using the ConSurf web-server (Glaser, F., et al, The ConSurf Server, http://consurf.tau.ac.il/, 2003. Date of access 07/11/2013). In the analysis, the empirical Bayesian calculation method and LG substitution matrix were chosen to infer the scores. The scores were automatically divided into nine successive equally sized categories and assigned to different color codes according to the relative degree of conservation: grade 1 contains the most variable positions and grade 9 contains the most conserved positions. Regions under heavy selection detecting by TreeSAAP were correlated back to the 3D structure of the *Bos taurus* UT-B complex using the program Pymol (Schrodinger, 2010). According to the evolutionary rate of each position of the protein and known mechanisms of urea conduction of dvUT and UT-B, the physicochemical evolutionary adaptation and potential mechanisms of UT-A2 were discussed.

## Author Contributions

Conceived and designed the experiments: D.W. and J.Z.W. Performed the experiments: J.Z.W., X.Y.Y. and B.H. Analyzed the data: J.Z.W. and X.Y.Y. Contributed reagents/materials/analysis tools: J.S.Z., W.H.X. and Y.J.H. Wrote the paper: J.Z.W., W.H.L. and D.W.

## Supplementary Material

Supplementary Information Physicochemical Evolution and Molecular Adaptation of the Cetacean Osmoregulation-related Gene UT-A2 and Implications for Functional Studies

## Figures and Tables

**Table 1:** Log likelihood values and parameter estimates of different models by PAML. 2Δℓ: double likelihood difference

Model	ln L	Estimates of parameters	Models compared	2Δℓ	P value	Sites positively selected
***Branch models***						
(A) M0: one-ratio	−3268.299851	ω = 0.08625				
(B) free ratio	−3249.845099	variable ω by branch:(Human0.0509,(Cow0.1447,((((Bottlenose_dolphin0.3848,Pilot_whale0.1284)0.5365,Finless_porpoise0.0001)0.0001,Baiji0.3906)0.0001,(Minke_whale0.7312,(Sei_whale0.0001,Brydes_whale0.0001)0.0991)0.2341)0.0911)0.0506,(Mouse0.1079,Rat0.0988)0.0558)	A *vs* B	88.318236	2.9×10^−11^**	
(C) all branches have same ω = 1	−3499.431148	ω = 1	A *vs* C	462.262594	1.5 ×10^−102^**	
(D) Two ratio (**branch cetacean**)	−3266.219683	ω0 = 0.07497ω1 = 0.13907	A *vs* D	4.160336	0.041*	
(E) neutral model (branch cetacean)	−3294.004217	ω0 = 0.0705ω1 = 1	D *vs* E	55.569068	9.0×10^−14^**	
(F) Two ratio (**Branch baiji**)	−3265.523929	ω0 = 0.08178ω1 = 0.39522	A *vs* F	5.551844	0.018*	
(G) neutral model (branch baiji)	−3266.393028	ω0 = 0.08171ω1 = 1	F *vs* G	1.738198	0.187	
(H) Two ratio (**Branch YFP**)	−3267.143786	ω0 = 0.08754ω1 = 0.00010	A *vs* H	2.31213	0.128	
(I) neutral model (branch YFP)	−3273.394515	ω0 = 0.08749ω1 = 1.00000	H *vs* I	12.501458	0.0004	
***Site models***						
M3(k = 3)	−3247.386350	p0 = 0.63514p1 = 0.33045p2 = 0.03441ω0 = 0.00000ω1 = 0.21227ω2 = 0.82386	M0 *vs* M3 (k = 3)	41.827002	1.8×10^−8^**	
M3(K = 2)	−3247.617742	p0 = 0.79369p1 = 0.20631ω0 = 0.01869ω1 = 0.39995	M0 *vs* M3 (k = 2)	41.364218	1.0×10^−9^**	
M7: beta	−3247.510906	p = 0.19794q = 1.73772				
M8: beta & ω	−3247.505476	p0 = 0.99513p = 0.20684q = 1.89985(p1 = 0.00487)ω = 1	M7: beta *vs* M8: beta & ω	0.01086	0.995	
M1a	−3251.362169	p0 = 0.93299p1 = 0.06701ω0 = 0.05006ω1 = 1				
M2a	−3251.362169	p0 = 0.93299p1 = 0.05530p2 = 0.01172ω0 = 0.05006ω1 = 1ω2 = 1	M1a *vs* M2a	0	1	
***Branch-site models (Cetacean)***						
Model A	−3246.391824	ω0 = 0.04693ω1 = 1.00000ω2 = 3.14931p0 = 0.92209p1 = 0.05334p2a = 0.02322p2b = 0.00134	M1a *vs* Model A	9.94069	0.007**	154, 264, 328
Model A null	−3247.455370	ω0 = 0.04365ω1 = 1ω2 = 1p0 = 0.88482p1 = 0.05550p2a = 0.0566p2b = 0.00352	Null model A *vs* model A	2.127092	0.144	
Model B	−3243.468657	ω0 = 0.02018ω1 = 0.39328ω2 = 2.86277p0 = 0.80077p1 = 0.17119p2a = 0.02310p2b = 0.00494	M3 (K = 2) *vs* Model B	8.29817	0.016*	154, 264, 328
***Branch-site models (Baiji)***						
Model A	−3247.955988	ω0 = 0.04428ω1 = 1.00000ω2 = 1.17759p0 = 0.68094p1 = 0.05149p2a = 0.24875p2b = 0.01881	M1a *vs* Model A	6.812362	0.033*	7, 149, 331
Model A null	−3247.956900	ω0 = 0.04425ω1 = 1ω2 = 1p0 = 0.63832p1 = 0.04833 p2a = 0.29129 p2b = 0.02205	Null model A *vs* model A	0.001824	0.966	
Model B	−3243.587111	ω0 = 0.01139ω1 = 0.37489ω2 = 0.62888p0 = 0.34035p1 = 0.09753p2a = 0.43693p2b = 0.12520	M3 (K = 2) *vs* Model B	8.061262	0.018*	
***Branch-site models (YFP)***						
Model A	−3251.362172	ω0 = 0.05006ω1 = 1.00000ω2 = 1.000p0 = 0.93299p1 = 0.06701p2a = 0.000p2b = 0.000	M1a *vs* Model A	6×10^−6^	0.9999	
Model A null	−3251.362169	ω0 = 0.05006ω1 = 1ω2 = 1p0 = 0.63832p0 = 0.93299p1 = 0.06701p2a = 0.000p2b = 0.000	Null model A *vs* model A	6×10^−6^	0.998	
Model B		ω0 = 0.01874ω1 = 0.40446ω2 = 0p0 = 0p1 = 0p2a = 0.79236p2b = 0.20764	M3 (K = 2) *vs* Model B	2.307064	0.315	

(‘**’: p < 0.01, highly significant; ‘*’: p < 0.05, significant).

**Table 2:** Sites of UT-A2 protein identified as being under positive, episodic directional or positive destabilizing selection by PAML, Consurf, DataMonkey and TreeSAAP programs

Codon	Branch or clade	AA change	Detected Method				Domain	AA property	
			ConSurf (Bayesian and LG)	Datamonkey (MEME)	PAML (MB-M3 and MA-M1a)	TreeSAAP		property	Category(+/−shift)
3	Baleen whale	Glu → Lys				**+**	N-terminus	pH_i_,	8(+6.52)
	Cow	Glu → Asp				**+**		P_t_	6 (+0.72)
7	Baiji	Ile → Val				**+**	N-terminus	pK′	8 (+0.96)
12	Marine toothed whale	Gly → Asp		**+**		**+**	N-terminus	E_t_	6(−0.38)
59	Minke whale	Asp → Gly				**+**	Pa	E_t_	6(+0.38)
65	Minke whale	Thr → Val	**+**	**+**		**+**	Pa	N_s_, R_α_	6(+1.81), 7(+4.54)
86	Cow	Val → Ile	**+**			**+**	T1a	pK′	8(−0.96)
100	Cetartiodactyla	Ile → Val	**+**			**+**	T2a	pK′	8(+0.96)
108	Baleen whale	Ile → Ser				**+**	T2a	N_s_, B_r_, R_F_, P_c_, pK′, F, R_α_, H_t_, P_t_	6(−1.98), 6(−0.33), 6(−10.2), 6(+0.68), 7(+0.85), 7(+0.37), 8(−5.26), 7(−3.08), 8(+0.96)
149	Baiji	Val → Ile				**+**	T4a	pK′	8(−0.96)
151	Bottlenose dolphin	Val → Ile	**+**			**+**	T4a	pK′	8(−0.96)
154	Cetacean	Met → Val	**+**		**+ +**		T4a		
	Bottlenose dolphin	Val → Met							
200	Cetacean	Thr → Lys	**+**			**+**	Apical loop	pH_i_, E_t_	6(+4.08), 6 (−0.4)
	Baiji	Lys → Thr						pH_i_, E_t_	6(−4.08), 6(+0.4)
205	Cetacean	Ala → Ser	**+**			**+**	Apical loop	P_α_, P_c_, P_t_	6(−0.65), 6(+0.63), 6(+0.77)
206	Rodentia	Ser → Ala	**+**			**+**	Apical loop	P_α_, P_c_, P_t_	6(+0.65), 6(−0.63), 6(−0.77)
	Mouse	Ala → Thr				**+**		P_α_,	6(−0.59)
	Rat	Ala → Val							
207	Cetartiodactyla	Ala → Ser	**+**			**+**	Apical loop	P_α_, P_c_, P_t_	6(−0.65), 6(+0.63), 6(+0.77)
208	Cow	Met → Val	**+**				Apical loop		
216	Cow	Val → Ile	**+**			**+**	Apical loop	pK′	8(−0.96)
264	Baleen whale	Thr → Ala	**+**		**+ +**	**+**	T2b	P_α_	6(+0.59)
	Marine toothed whale	Thr → Ala				**+**			
265	Cow	Met → Ile	**+**			**+**	T2b	pK′	8(−0.92)
312	Cetacean	Ile → Val				**+**	T4b	pK′	8(+0.96)
328	Cetacean	Met → Val	**+**		**+ +**	**+**	T4b		
	Pilot whale	Val → Ile				**+**		pK′	8(−0.96)
376	Cow and Rodentia	Tyr → His	**+**			**+**	C-terminus	F	4(−0.21), 6(−0.32)

Abbreviations: **P_α_**, alpha-helical tendencies; **N_s_**, average number of surrounding residues; **B_r_**, buriedness; **R_F_**, chromatographic index; **P_c_**, coil tendency; **pK**′, equilibrium constant-ionization of COOH; **pH_i_**, isoelectric point; **F,** mean r.m.s. fluctuation displacement; **R_α_**, solvent accessible reduction ratio; **H_t_**, thermodynamic transfer hydrophobicity; **E_t_**, total non-bonded energy; **P_t_**, turn tendencies.

**Table 3:** Summary of physicochemical amino acid properties detected for positive-destabilizing selection in this study

Property	Description
**P_α_**	Decreases in α-helical tendency broadens the outer surface area of a helix, and allows a more flexible surface area contact with neighboring protein constituents, whereas increases have the opposite effect^79^.
**N_s_**	N_s_ is number of residues surrounding a residue within the effective distance of influence^73^.
**B_r_**	Buried nature of residues within a globular protein^73^.
**R_F_**	R_F_ is characteristic migration rate in a solvent-absorbent system^73^.
**Pc**	The probability of a residue being in a coil^73^.
**pK′**	pK′ is a biochemical amino acid property that is a measure of the net charge in the overall charge resulting from the ionization of carboxyl groups^73^.
**pH_i_**	“pHi…the isoionic point of the free amino acid…includes the ionizable character of either the sidechain or amino group plus the carboxyl group of the molecule…”^105^.
**F**	This parameter relates to the amount of displacement of any element with its distance from the centroid of the protein. Each protein is characterized by its specific internal residue-distribution and associated fluctuational situation^106^.
**V^0^**	Partial specific volume is the reciprocal of density^73^.
**E_sm_**	Sum of all non-bonded energy between atoms within ten residues along a polypeptide chain^73^.
**R_α_**	Ratio of accessibility of water “from a hypothetical extended state to the native fold state”, the increase in this value suggest that proteins could have become bulkier and allowing more space for active site formation^73^.
**H_t_**	“---intrinsic hydrophobic character of the (amino acid) side chain---”^73^.
**E_t_**	Sum of short-, medium-, and long-range non-bonded energies^73^,^74^.
**P_t_**	P_t_ refers to the ability of a specific amino acid to contribute to or initiate a turn^73^.
