# Supplementary material for: Physicochemical Evolution and Molecular Adaptation of the Cetacean Osmoregulation-related Gene UT-A2 and Implications for Functional Studies
Source: Sci Rep. 2015 Mar 12;5:8795. doi: 10.1038/srep08795 (PMC4357013; doi:10.1038/srep08795)
Supplement: Supplementary Information — Physicochemical Evolution and Molecular Adaptation of the Cetacean Osmoregulation-related Gene UT-A2 and Implications for Functional Studies [file srep08795-s1.pdf]

Physicochemical Evolution and Molecular Adaptation of the Cetacean Osmoregulation-related Gene UT-A2 and Implications for Functional Studies

Jingzhen Wang[1,2&], Xueying Yu[1,3&], Bo Hu[1], Jinsong Zheng[1], Wuhan Xiao[1], Yujiang Hao[1], Wenhua Liu[2*], Ding Wang[1,*]

[1] Key Laboratory of Aquatic Biodiversity and Conservation of the Chinese Academy of Sciences; Institute of Hydrobiology, Chinese Academy of Sciences, Wuhan, Hubei 430072, China

[2] Marine Biology Institute, Shantou University, Shantou, Guangdong 515063, China

[3]University of Chinese Academy of Sciences, Beijing 100039, China

*To whom correspondence should be addressed:

Wenhua Liu, Ph.D., Shantou University, Shantou, Guangdong 515063, P. R. China. Fax: +86-754-86500614; E-mail: whliu@stu.edu.cn

Ding Wang, Ph.D., Institute of Hydrobiology, Chinese Academy of Sciences, Wuhan, Hubei 430072, P. R. China. Fax: +86-27-68780123; E-mail: wangd@ihb.ac.cn

[&] These authors contributed equally to this work

Table S1 Primers used for RT-PCR amplification of Yangtze finless porpoise UT-A2 gene.

Primers	Sequence (5′ to 3′)	Position
F1	ATGGAGRAGAGCTCYGAGATAAA	1-23
R1	TTAYGATGACGGGGARTAGAAGC	432-454
F2	TTCTCRGACAAAGGCGACTAYTA	403-425
R2	GTCAGGAGCAGGAAGGTGAGKG	1034-1055
F3	GCTTTTGAGAGCCATCCCTGTW	660-681
R3	TTAGGASACATCGTAGGCCTGGTA	1171-1194

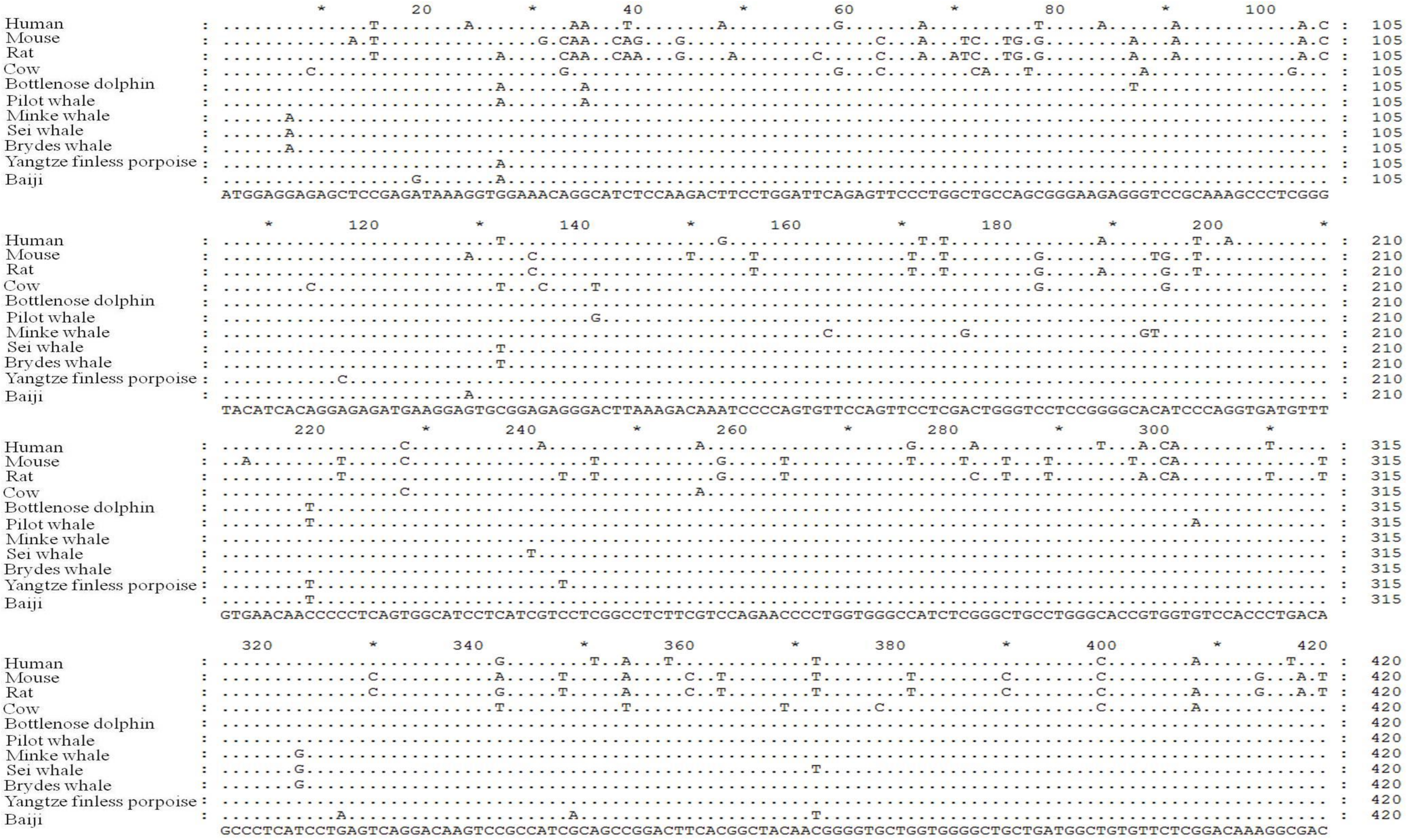
Human
Mouse
Rat
Cow
Bottlenose dolphin
Pilot whale
Minke whale
Sei whale
Brydes whale
Yangtze finless porpoise
Baiji
ATGGAGGAGAGCTCCGAGATAAAGGTGGAAACAGGCATCTCCAAGACTTCCTGGATTCAGAGTTCCCTGGCTGCCAGCGGGAAGAGGGTCCGCAAAGCCCTCGGG
TACATCACAGGAGAGATGAAGGAGTGCGGAGAGGGACTTAAAGACAAATCCCCAGTGTTCCAGTTCCTCGACTGGGTCCTCCGGGGCACATCCCAGGTGATGTTT
GTGAACAACCCCCTCAGTGGCATCCTCATCGTCCTCGGCCTCTTCGTCCAGAACCCCTGGTGGGCCATCTCGGGCTGCCTGGGCACCGTGGTGTCCACCCTGACA
GCCCTCATCCTGAGTCAGGACAAGTCCGCCATCGCAGCCGGACTTCACGGCTACAACGGGGTGCTGGTGGGGCTGCTGATGGCTGTGTTCTCGGACAAAGGCGAC

```
                               *      440         *      460         *      480         *      500         *      520
Human                   : ..C...........GT.G..A.........A.C......A...............................T..........................A...... : 525
Mouse                   : ..C...........G..G..T..T.....T..C......A..A.............T............A.....G......................A...... : 525
Rat                     : ..C...........G..G.....G........C......A..A......................T...A.....G..........A...........A...... : 525
Cow                     : ....T..................G...............A................................................................. : 525
Bottlenose dolphin      : ..............................A.A......A................................................................. : 525
Pilot whale             : ................................A........................................................................ : 525
Minke whale             : ......................................................................................................... : 525
Sei whale               : ......................................................................................................... : 525
Brydes whale            : ......................................................................................................... : 525
Yangtze finless porpoise: ................................A........................................................................ : 525
Baiji                   : ........................A................................................................................ : 525
                          TATTACTGGTGGCTTCTACTCCCCGTCATCGTGATGTCCGTGTCTTGCCCCATCCTCTCCAGTGCCCTGGGCACCATCTTCAGCAAGTGGGACCTCCCGGTCTTC

                              *      540         *      560         *      580         *      600         *      620         *
Human                   : ..A...........T...A.T.....TT...............................T...........A.C............TG.A...G..........C : 630
Mouse                   : ..A.................C....................G.........C....T................C......A.....TA.AA..A.TCCA.....C : 630
Rat                     : ..A.................C...................................T.............................TG.AGTTA...CA.....C : 630
Cow                     : ..C.................C.................G..................................CA...........GGT.......G.......C : 630
Bottlenose dolphin      : ......................................G.....A............................................................ : 630
Pilot whale             : ......................................G.................................................................. : 630
Minke whale             : ......................................................................................................... : 630
Sei whale               : ................................................................................................T........ : 630
Brydes whale            : ................................................................................................T........ : 630
Yangtze finless porpoise: ......................................G.................................................................. : 630
Baiji                   : .........................................................................C....G.......................... : 630
                          ACGCTGCCCTTCAACATCGCAGTGACCCTGTACCTGGCAGCCACGGGCCACTACAACCTCTTCTTCCCCACGAAGCTGCTGCAGCCCTCCTCCTCCATGCCCAAT

                                 640         *      660         *      680         *      700         *      720         *
Human                   : ...........A...................................C........T.................T.......................C...... : 735
Mouse                   : ...........A..TA........T.T......C.............G.......................G..T..C....................C.....G : 735
Rat                     : ...........A..T...........T......C.............C....................T.....T..C..T.................C...... : 735
Cow                     : ...........A...A......................................................................................... : 735
Bottlenose dolphin      : .....G................................................................................................... : 735
Pilot whale             : ......................................................................................................... : 735
Minke whale             : ......................................................................................................... : 735
Sei whale               : ......................................................................................................... : 735
Brydes whale            : ......................................................................................................... : 735
Yangtze finless porpoise: ......................................................................................................... : 735
Baiji                   : ......................................................................................................... : 735
                          ATCACCTGGTCGGAGGTCCAAGTGCCCTTGCTTTTGAGAGCCATCCCTGTTGGAATCGGCCAAGTGTACGGCTGCGATAACCCCTGGACTGGAGGCATTTTCCTC

                             740         *      760         *      780         *      800         *      820         *      840
Human                   : ......C..........C........C..............A..A..T..................C.......A......A.T.....G............... : 840
Mouse                   : G.C...C.......C..T..G.....C.................C..T...........A..............A....GCA.T..C.....A............ : 840
Rat                     : G.T...C.....G.C..T........C..C........C.....C...........G..A..............A....GCA.T..C.....A............ : 840
Cow                     : .................A..G.......................A..T...........T..............T...........A..G............... : 840
Bottlenose dolphin      : ......................................................G.................................................. : 840
Pilot whale             : ......................................................G.................................................. : 840
Minke whale             : ......................................................G.................................................. : 840
Sei whale               : ............................................A.........G............................G..................... : 840
Brydes whale            : ............................................A.........G............................G..................... : 840
Yangtze finless porpoise: ......................................................................................................... : 840
Baiji                   : ......................................................................................................... : 840
                          ATAGCTTTGTTCATATCGTCACCTCTTATTTGCTTGCATGCTGCGATCGGATCCACCATGGGGATGTTAGCAGCCCTCACTCTCGCGACACCCTTTGACTCCATC
```

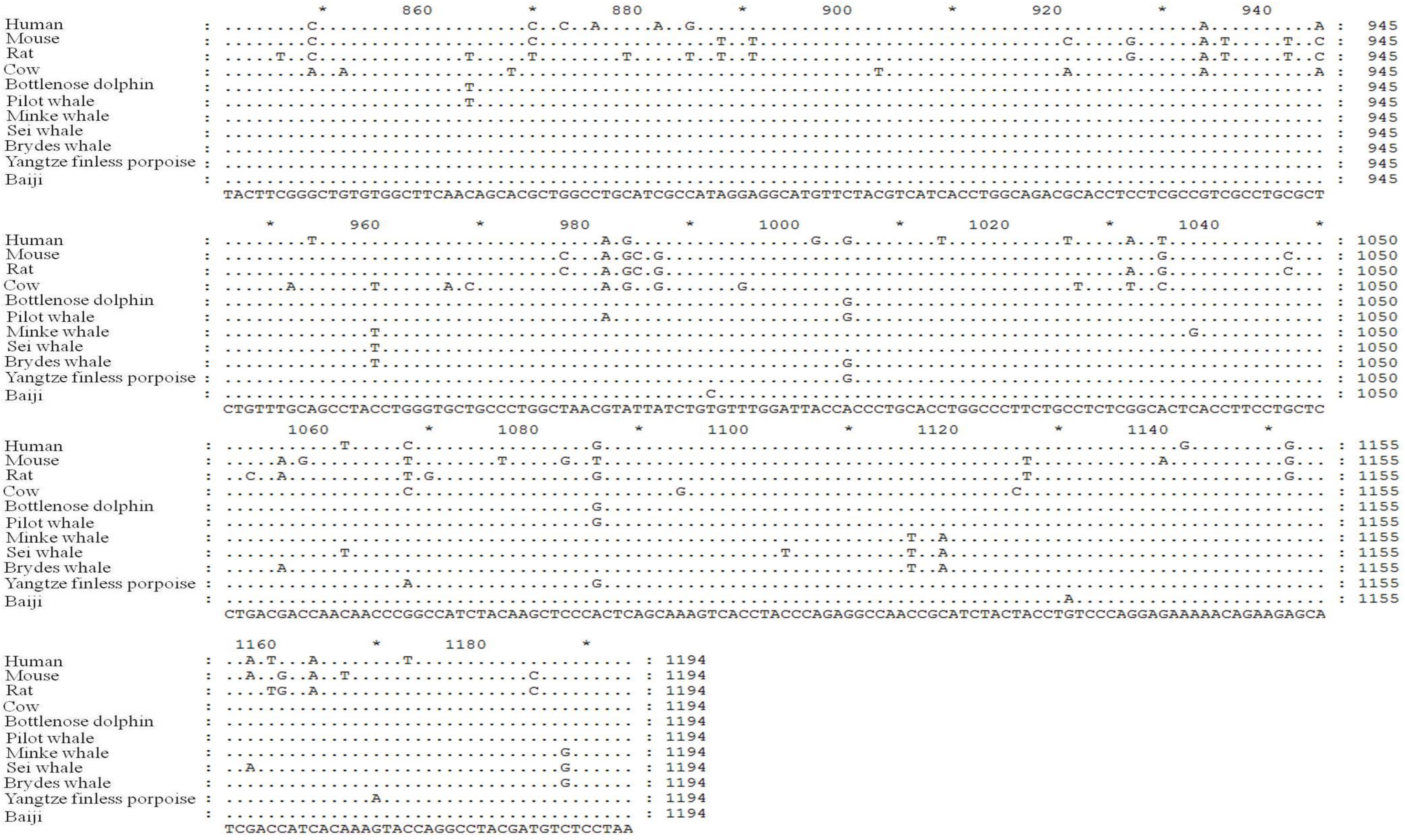
Human
Mouse
Rat
Cow
Bottlenose dolphin
Pilot whale
Minke whale
Sei whale
Brydes whale
Yangtze finless porpoise
Baiji
860 880 900 920 940
945
TACTTCGGGCTGTGTGGCTTCAACAGCACGCTGGCCTGCATCGCCATAGGAGGCATGTTCTACGTCATCACCTGGCAGACGCACCTCCTCGCCGTCGCCTGCGCT
960 980 1000 1020 1040
1050
CTGTTTGCAGCCTACCTGGGTGCTGCCCTGGCTAACGTATTATCTGTGTTTGGATTACCACCCTGCACCTGGCCCTTCTGCCTCTCGGCACTCACCTTCCTGCTC
1060 1080 1100 1120 1140
1155
CTGACGACCAACAACCCGGCCATCTACAAGCTCCCACTCAGCAAAGTCACCTACCCAGAGGCCAACCGCATCTACTACCTGTCCCAGGAGAAAAACAGAAGAGCA
1160 1180
1194
TCGACCATCACAAAGTACCAGGCCTACGATGTCTCCTAA

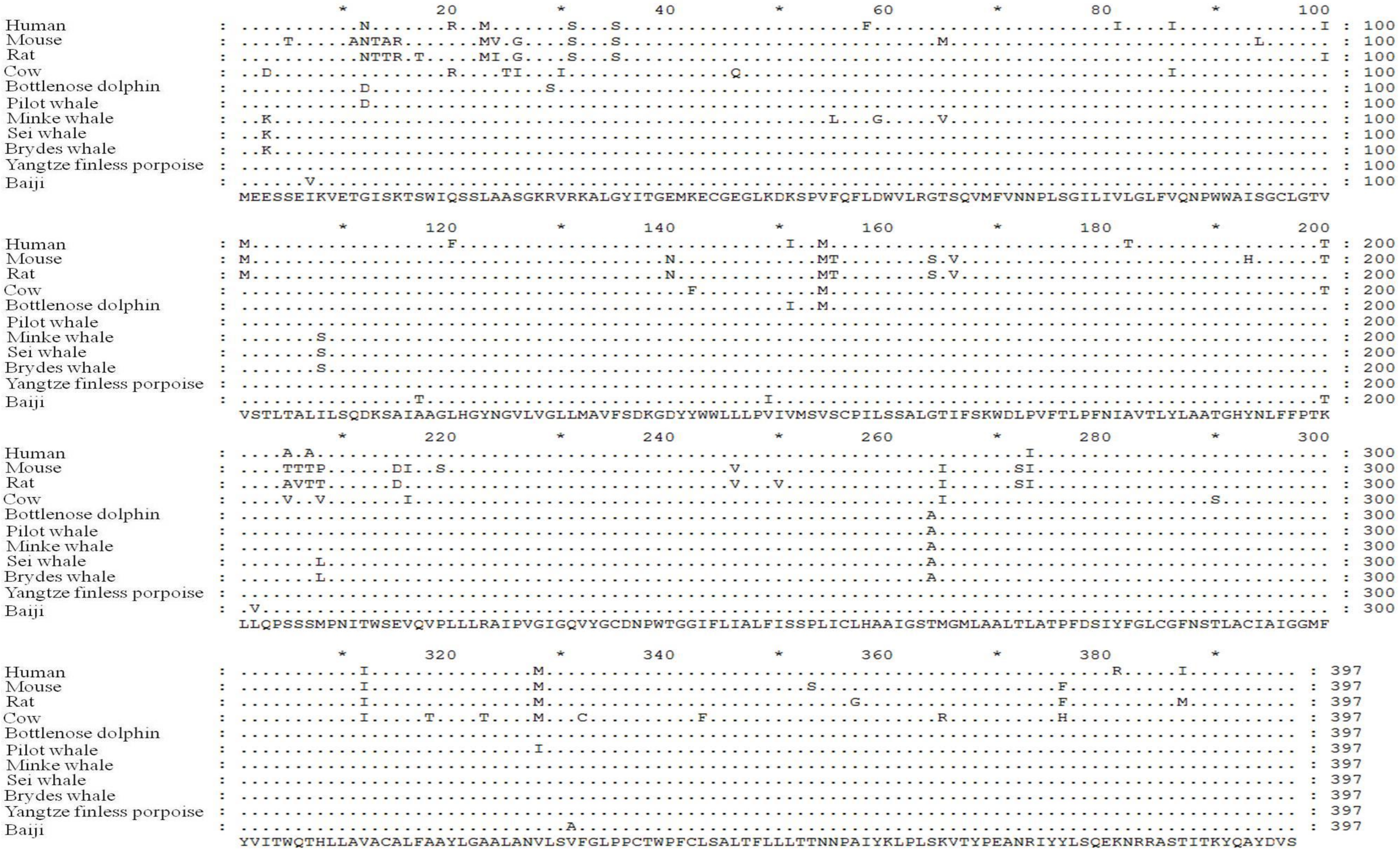

Figure S1 Alignments of CDS and amino acids sequences of gene UT-A2.
